# The Global Economic Cost of Osteoarthritis: How the UK Compares

**DOI:** 10.1155/2012/698709

**Published:** 2012-10-02

**Authors:** A. Chen, C. Gupte, K. Akhtar, P. Smith, J. Cobb

**Affiliations:** MSK Lab, Imperial College, London W6 8RF, UK

## Abstract

*Aims*. To examine all relevant literature on the economic costs of osteoarthritis in the UK, and to compare such costs globally. *Methods*. A search of MEDLINE was performed. The search was expanded beyond peer-reviewed journals into publications by the department of health, national orthopaedic associations, national authorities and registries, and arthritis charities. *Results*. No UK studies were identified in the literature search. 3 European, 6 North American, and 2 Asian studies were reviewed. Significant variation in direct and indirect costs were seen in these studies. Costs for topical and oral NSAIDs were estimated to be **£**19.2 million and **£**25.65 million, respectively. Cost of hip and knee replacements was estimated to exceed **£**850 million, arthroscopic surgery for osteoarthritis was estimated to be **£**1.34 million. Indirect costs from OA caused a loss of economic production over **£**3.2 billion, **£**43 million was spent on community services and **£**215 million on social services for osteoarthritis. *Conclusions*. While estimates of economic costs can be made using information from non-published data, there remains a lack of original research looking at the direct or indirect costs of osteoarthritis in the UK. Differing methodology in calculating costs from overseas studies makes direct comparison with the UK difficult.

## 1. Introduction

Musculoskeletal diseases remain one of the most common causes for severe long-term pain and disability. The increasing significance of musculoskeletal disorders has prompted the United Nations, the World Health Organization, and 37 countries to spearhead a campaign to recognise and address the burden of musculoskeletal disorders such as arthritis, proclaiming it to be the *Bone and Joint Decade *(2000–2010) [1], and to advance understanding and treatment of musculoskeletal disorders through prevention, education, and research.

Within the envelope of musculoskeletal disorders, Osteoarthritis represents a complex musculoskeletal disorder with multiple genetic, constitutional, and biomechanical risk factors. It represents the most common form of joint disease and disability in older people and ranks amongst the top 5 causes of disability [2]. 

The economic costs of osteoarthritis can be broken down into direct costs and indirect costs. Direct costs represent the pharmacological/nonpharmacological treatments, including surgery, as well as use of hospital resources and management of complications arising from the treatment of osteoarthritis. Indirect costs represent loss of time from work, decreased productivity because of pain, care-giver time, premature mortality, and disability compensation/benefits. These costs are summarised in Table 1, below.

A third category sometimes considered is that of intangible costs. These are defined as the pain and suffering experienced by the patient as a result of the disease; the reduction in the patient's quality of life. They remain an area of controversy, with only a few studies making the attempt to estimate them [3]. 

## 2. Aims

The aim of this paper is to examine all relevant literature on the economic costs of Osteoarthritis in the UK, and to see what comparisons can be made regarding such costs in the UK and other countries in North America, Europe, and Asia.

## 3. Methods

A comprehensive review of the literature was performed using a computerised bibliographical search of MEDLINE databases from 1946 to 31st Dec 2011. English language articles were reviewed that contained the words “economic cost,” “direct cost,” or “indirect cost” in combination with “osteoarthritis” in either their title or abstract. 

To expand the review beyond only published studies, an internet search was made for publications from the UK Department of Health, the National Institute for Clinical Excellence, the UK National Joint Registry, Hospital Episode Statistics, and charities Arthritis Research (UK) and Arthritis Care, and all publications were reviewed for information on costs for osteoarthritis. Further internet searches were made for publications from the British Orthopaedic Association, the Royal College of Surgeons, the Royal College of Physicians, and the Royal College of General Practitioners. Publications from American Association of Orthopaedic Surgeons and other regional orthopaedic associations in Europe and Asia were also reviewed.

## 4. Results

### 4.1. What Do We Know about OA Costs in North America?

Studies on prevalence of osteoarthritis in the United States have shown that osteoarthritis affects 13.9% of adults aged 25 and older, and 33.6% of those over the age of 65, with an approximate 27 million Americans of all ages suffering from disease [4]. 

Much of the data available on osteoarthritis in the United States is derived from studies conducted in the 1960s and 1970s. The Framingham study [5] represented one of the early studies to associate increasing age with worsening knee arthritis. This study, which began in 1948, was initially designed to look at cardiovascular risk factors in a representative sample of people in the adult population of Framingham, MA. The study's patients were examined every 2 years since inception. This same cohort of patients was used by Felson et al. to look at the prevalence of knee osteoarthritis approximately 36 years after the start of the study. The age of the patients in the study ranged from 63 to 94, and a total of 1805 patients were studied. The study confirmed that radiographic evidence of OA increased with age, with a higher prevalence of OA changes in women, as well as a significantly higher proportion of women with symptomatic OA.

Lethbridge-çejku et al. [6] examined discharge data from the National Hospital Discharge Survey and concluded that Osteoarthritis accounts for 55% of all arthritis related hospital admissions, with 409 000 such admissions in 1997. The annual cost of knee and hip replacements in 1997 was estimated at $7.9 billion (*£*4.7 billion) [6]. Less than 10 years later in 2004, the number of hospital admissions had risen to 632 000 and the annual total cost of joint replacements rose to $22.6 billion (*£*13.8 billion) [7]. 

Buckwalter et al. [8] used data drawn from national data sets collected by the U.S. Bureau of Labor Statistics, the U.S. National Center for Health Statistics, as well as existing cost estimates for arthritis in the literature, used proportional attributable risk models and the human capital method to break down costs into direct and indirect costs. From this study, an estimated $3.4–$13.2 billion (*£*2 billion–*£*8 billion) is spent annually on job-related OA costs in the USA. Meanwhile, Kotlarz et al., in 2010, using evidence from the national health survey data from 1996–2005, looked at absenteeism as a result of osteoarthritis. This study, estimated the indirect cost of the absenteeism to be approximately US$10.3 billion [9]. The study also confirmed that the costs for women were larger (US$ 5.5 billion compared to US$4.8 billion), and that absenteeism was less in subjects with lower education and those in minority groups.

A survey done by Gupta et al, in ON, Canada in 2005, estimated that the indirect costs incurred by a patient aged over 55 with hip or knee arthritis may be much higher than previously estimated when compared to direct costs ($12 990 or *£*8183 annually for the former, and $2300 or *£*1449 for the latter) [10]. Indirect costs were incurred mostly for time lost from employment and for unpaid informal caregivers, with caregiver time accounting for 40% of indirect costs. It should be noted that the authors in this study based the costs on the monthly wage for a professional homemaker or housekeeper as caregiver occupation was unknown. This averaged at US$ 1278 (*£*824) per month, which may explain the relatively higher costs reported. 

March and Bachmeier (1997) looked at the global cost of osteoarthritis and found the cost of osteoarthritis in the USA, Canada, UK, France, and Australia to account for between 1–2.5% of the gross national product (GNP) for those countries [11]. 

### 4.2. How Do Recent Studies in Europe Compare?

Loza et al., in their Spanish study, assessed the burden of knee and hip osteoarthritis by examining 1071 patients across all the provinces of Spain [12]. The average annual cost for OA per patient was estimated at €1502 (*£*1260), with direct costs representing 86% (*£*1084) of the total cost. Indirect costs were much lower (14% or *£*176) and mainly involved domestic help.

In contrast to this, the COART study [13] in France attempted to estimate the overall financial cost of osteoarthritis to the country. The study concluded that osteoarthritis remained a major public burden, with direct costs in 2002 exceeding 1.6 billion Euros, about 1.7% of the expenses of the French Health system. Over 13 million visits were made to physicians for osteoarthritis. Medication costs were 570 million euros and inpatient treatment amounted to 820 million Euros. During the period of the study, 80 000 total hip replacements and 38 000 total knee replacements were performed per year, at a cost of €5600 per THR and €4500 per TKR. The study compared the costs to a previous study by Levy et al. [14] in 1993 and found that the prevalence of the disease had risen by 54%, and the direct medical costs by 156%.

In Italy, Leardini et al. examined the economics of osteoarthritis of the knee in 2004. They used a bottom-up method, utilising data collected from each patient, and reviewed patients across 29 medical institutes. They concluded that the direct costs came to €934 (*£*785) and indirect costs €1236 (*£*1039) per patient per year [15].

### 4.3. Is the Situation Different in Asia?

There have been fewer studies with regard to economic costs of osteoarthritis in India, China or Southeast Asia compared with countries in the western hemisphere. 

In contrast to the Western literature, Woo et al., in their Hong Kong [16] study estimated that the cost of osteoarthritis accounted for 0.28% of the GNP of Hong Kong, between HK $3.2–$3.9 billion (*£*253 million–*£*308 million). The direct costs ranged from HK $4860–$11180 (*£*384–*£*883) and indirect costs HK$3300–$6640 (*£*261–*£*525) per person annually.

Xie et al. assessed indirect costs in Singapore for OA and noted that there were estimated at between US$1000–1200 (*£*610–*£*730), around 2.8% and 3.3% of the annual household income [3]. The authors here acknowledged that these costs likely represented the lower end of the scale, as costs such as loss of productivity of caregivers were not estimated. The study was also one of the few that attempted to address and estimate intangible costs using the willingness to pay (WTP) method. In economics, this model represents the maximum amount a person would be willing to pay, sacrifice, or exchange in order to avoidsomething undesired, in this case, the pain and suffering associated with osteoarthritis. The authors here estimated the intangible costs at US$ 1200 (*£*775) per year.

### 4.4. What Do We Know about the Economic Cost of OA in the UK?

#### 4.4.1. The Prevalence of OA

The Arthritis Research Council (UK) estimated in 2002 that at least 4.4 million patients in the UK have X-ray evidence of moderate-to-severe osteoarthritis in their hands, while 550 000 have similar evidence of osteoarthritis in their knees, and 210 000 have evidence of this in their hips [17].

Pye et al., in 2004, showed that almost 8.5 million people in the UK have X-ray evidence of osteoarthritis in their spine, with back pain being the most frequent symptom [18]. While predominantly a disease of the elderly, an estimated 6% of adults aged 30 and above have both knee pain and radiographic changes of osteoarthritis [18].

The Royal College of General Practitioners estimated in 2006 that in the UK over 1 million adults consult their GP each year with symptoms of osteoarthritis [19]. Another study in 2007 showed that consultations for osteoarthritis account for 15% of all musculoskeletal consultations in those aged 45 and over, rising to 25% in those aged 75 and over [20]. The cost per consultation is estimated at *£*36 for a 12-minute consultation [21].

During the year from 1999-2000, there were 114,500 hospital admissions related to osteoarthritis in the UK [17]. The latest Hospital Episode Statistics (HES) data (2010-2011) have shown a significant increase in hospital admissions, for hip and knee arthritis alone, the combined figure was 181,350 admissions [20]. When the diagnoses for polyarthritis and “other arthritis”–but not rheumatoid conditions or crystal arthropathy are included, the total number of admissions in 2010/11 was 207,041, representing an 80% increase compared with figures of 10 years ago.

Surprisingly, there are no published studies in the literature with regard to direct or indirect costs of osteoarthritis in the UK. Data, however, is instead only available from a variety of other sources. 

### 4.5. What Information Is Available from Other Sources about Costs in the UK?

#### 4.5.1. Direct Costs

The National Institute of Clinical Excellence (NICE) recently published a costing report in 2008 with regard to implementing the guidelines for treatment of osteoarthritis [22]. In the report, NICE estimated the prevalence of osteoarthritis in the UK to be a total of 2.8 million patients, based on symptomatic diagnosis in patients aged over 45. The analysis covered the management of osteoarthritis in all such patients.

The cost of topical and oral nonsteroidal anti-inflammatories (NSAIDs) was estimated using prescribing data from 2005/06 [22]. An estimated 167 000 people who had a diagnosis of osteoarthritis were found to have been prescribed topical NSAIDs, and it was estimated that 50% (1.4 million patients) of patients with osteoarthritis were prescribed oral NSAIDs. The annual cost in 2005/06 of prescribing topical NSAIDs was *£*8.5 million and *£*25 million for oral NSAIDs. The cost for topical NSAID prescriptions was anticipated to double, and the cost of oral treatment reduced by 10%, if the new guidelines are followed. Adjusting for inflation, in 2010 prices, this would equate to *£*19.2 million and *£*25.65 million, respectively.

The cost of iatrogenic events related to NSAID use is also substantial. NSAID-related iatrogenic events have been estimated to be between *£*32–*£*70 per patient prescribed an NSAID in the UK. This equates to a total cost of *£*44.8–*£*98 million per year (*£*56.9–*£*124.4 million at 2010 prices) [23].

The cost of proton pump inhibitor (PPIs) prescription for use with NSAID treatment was *£*26 000 in 2005/06 but expected to rise significantly to *£*10.5 million (*£*11.6 million in 2010) with implementation of the new guidance. 

The 2005/06 Hospital Episode statistics stated that the total number of people aged 45 and over who received arthroscopic lavage and debridement for knee osteoarthritis was approximately 20 000. The national tariff for arthroscopies set by the Health Resource Group in 2008/09 was *£*1264, resulting in a cost *£*25 million for such treatment. It should be noted that NICE expected the cost of this to fall dramatically (by 19000 patients), with guidelines restricting the use of arthroscopic treatment to patients with “mechanical” symptoms such as locking or giving way. The new cost for arthroscopic treatment of osteoarthritis is calculated (*£*1264 × 1000 patients) at *£*1.26 million (*£*1.3 million in 2010). 

#### 4.5.2. Economics of Joint Replacement

According to the 8th annual report of the NJR, published September 2011 [22], a 76 759 primary total hip replacements were performed in 2010, a 6% increase from 2009. The revision “burden” was approximately 11% with 7852 hips revised in 2010. A total of 81979 knee replacements were done in 2010, representing an increase of 5.7% when compared with 2009. The revision “burden” here was less, at just over 6% requiring revision in 2010. The proportion of total knee replacements to unicondylar knee replacements and patella-femoral knee replacements have remained largely the same for the last few years. 

While there are other causes for joint replacement surgery, osteoarthritis remains the most frequent cause for hip replacement (93% of primary hip replacements in 2010) and knee replacement (97% of primary knee replacements in 2010). 

**Figure 1:**
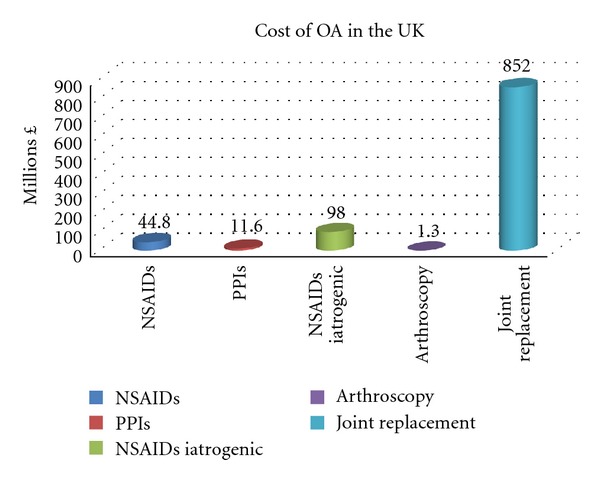


The costs of hip and knee replacements vary considerably from trust to trust in the UK with no set national price for implants, and the cost also being significantly dependant on length of hospital stay. The tariff reimbursement paid to the trust in one study [24] in 2005/06 was *£*6000 for a primary total hip replacement and *£*6800 for a primary total knee replacement. The national tariff for 2010 was set at *£*5552 for an uncomplicated total hip replacement and *£*5198 for a similar total knee replacement. This leads to an estimated cost of *£*426 million for total hip replacements and *£*426 million for primary total knee replacements, giving a combined total cost for primary hip and knee replacements of *£*852 million in 2010. This represents a substantial increase in costs over the last 10 years, when compared to the expenditure of *£*405 million in 2000 for 44 000 hip and 35 000 knee replacements [17]. Even adjusting for inflation, this cost would only be *£*514 million in 2010, representing a 66% increase in the last 10 years.  Figure 1 summarises the UK direct costs.

#### 4.5.3. Indirect Costs

Osteoarthritis has a significant negative impact on the UK economy with an estimated total cost of 1% of GNP [25]. The Department of Work and Pensions estimates that 36 million work days were lost because of osteoarthritis in 2002, resulting in a loss of economic production over *£*3.2 billion; while at the same time, *£*43 million was spent on community services and *£*215 million spent on social services for osteoarthritis [26]. 

Arthritis remains the most common condition for people to receive the Disability Living Allowance (DLA), with *£*2.41 billion paid to people claiming incapacity benefit due to arthritis and related conditions in 2001 [26]. More than half a million people receive the DLA because of arthritis, more than the total for heart disease, stroke, chest disease, and cancer combined [16]. Only around 1 in 200 of those on benefit later returns to work [27]. 

The most recent review of disability costs in the UK was done by Dame Carol Black [28] in her review of the health of the working age population. Unfortunately, the review did not offer a breakdown of the components in musculoskeletal disability costs, and so, despite the report, the exact contribution of OA to such costs in the UK remains unknown.

## 5. Discussion 

The review demonstrates that osteoarthritis represents an increasing economic burden to all countries, both from direct costs and indirect costs. Economic data on osteoarthritis has been made difficult because of problems defining the prevalence and incidence of the disease. There is only sparse literature available regarding economic costs in the east, but what is more surprising is the lack of clear costing studies in the west, especially in the UK. 

Direct costs may vary from country to country, which is to be expected given their different health systems, and even between institutions in a country. Significant variability is seen from these studies, making direct comparison difficult. Compounding the problem is the fact that the methodology used in estimating these costs can vary from study to study, and not all studies give a clear breakdown of the calculation of the direct costs involved [29]. 

Furthermore, in the studies that provide a breakdown of the direct costs, few include the cost of alternative therapies in the treatment of osteoarthritis. There is evidence that nearly half (47%) of older patients in one American study [30] used an alternative type of therapy, and these costs are considerable (US$ 1127 or *£*723 per annum). Hence, the true economic burden of direct costs in osteoarthritis is likely to be significantly higher than most of these studies indicate.

The significant variability in indirect costs from these studies is also a concern. This is likely to be due to the lack of a standardized method to estimate indirect costs–unfortunately, there remains at present no good evidence to support one preferred method over the others [29]. Most studies conclude that indirect costs, however, represent a largely underestimated economic burden to country, and as such, these estimates may just be the tip of the iceberg.

## 6. Conclusion

Our review of the literature suggests that while there are a large number of studies on economic costs of osteoarthritis, from multiple countries, the information available in the literature remains patchy and difficult to interpret. Some studies focus on the macroeconomic angle, looking at costs at a national scale or costs per capita while others focus on costs from the view of the individual patient with OA. Other studies are only specific for arthritis of a single joint. Even with studies compatible from this point of view, the varied methodology and lack of standardization of costing make it impossible to accurately compare economic costs, whether direct or indirect. These studies are summarized in Table 2.

Despite such difficulties, one conclusion does seem clear from these studies: that such costs are very substantial and are continuing to rise.

The continuing lack of published data regarding direct and indirect OA costs in the UK, especially from the patient perspective, shows that more research into this area is vital. This will allow us to fully appreciate the healthcare burden of OA in the UK, as well as to make more accurate financial planning for the provision for healthcare services for the treatment of OA in the subsequent decade to come.

## Figures and Tables

**Table 1:** 

Direct costs	Indirect costs	Intangible costs
Costs of surgery	Loss of productivity	Pain and suffering
Hospital resources	Absenteeism	Decreased quality of life
Caregiver time	Premature mortality	Potential depression/anxiety
Pharmacological and nonpharmacological treatment	Disability payments/benefits	
Costs of side effects from treatments		
Research		

**Table 2:** 

Author	Year of study	Country	Cost studied	Individual cost per annum (2010 *£*) per OA patient	Population cost per annum (2010 *£*)
McClean et al.	1993	USA	Direct costs	*£*1526	US $548 million
Lanes et al.	1994	USA	Direct costs	*£*496	N/A
Buckwater et al.	2000	USA	Indirect costs	N/A	*£*2 billion–*£*8 billion
Kotlartz et al.	2005	USA	Indirect costs	*£*355	*£*7.25 billion
Maetzel et al.	2000	Canada	Direct costs Indirect costs	*£*3162 *£*1407	N/A N/A
Gupta et al.	2002	Canada	Direct costs Indirect costs	*£*1768 *£*9986	N/A N/A
Loza et al.	2003	Spain	Direct costs Indirect costs	*£*1292 *£*209	*£*4.04 billion *£*654 million
Le Pen et al.	2003	France	Direct costs	*£*316	*£*1.58 billion
Leardini et al.	2001	Italy	Direct costs Indirect costs	*£* 981 *£*1299	N/A N/A
Woo et al.	2001	Hong Kong	Direct costs Indirect costs	*£*6561 *£*620	*£*323 million (combined cost)
Xie et al.	2005	Singapore	Indirect costs	*£*610–*£*730	N/A
